# Expanding the Use of Continuous Glucose Monitoring in Type 2 Diabetes Mellitus: Impact on Glycemic Control and Metabolic Health

**DOI:** 10.3390/life15101543

**Published:** 2025-10-01

**Authors:** Mi-Joon Lee, Bum-Jeun Seo, Jae-Hyoung Cho

**Affiliations:** 1 Department of Medical Information, Kongju National University, 56 Gongjudaehak-ro, Gongju-si 32588, Republic of Korea; mijoon1004@kongju.ac.kr; 2 Department of Internal Medicine, Seoul St. Mary’s Hospital, College of Medicine, The Catholic University of Korea, Seoul 06591, Republic of Korea; drhopper@catholic.ac.kr

**Keywords:** diabetes mellitus, digital health, continuous glucose monitoring, blood glucose, health behavior

## Abstract

This study aims to investigate the effects of continuous glucose monitoring (CGM) on glycemic control in patients with diabetes mellitus (DM) and to identify the sociodemographic or health behavioral factors that influence the outcomes. The data were collected from 510 diabetic patients prescribed to use CGM for 12 weeks and analyzed using SPSS 27.0. Paired samples *t*-tests were used to compare the glycemic control (HbA1c and fasting glucose) and metabolic health (body mass index and total cholesterol) measures of subjects before and after the CGM use, and independent *t*-tests were conducted to examine whether the effectiveness of CGM differs according to subjects’ sociodemographic and health behavioral characteristics. As a result of this study, the use of CGM resulted in a significant reduction in HbA1c from 8.09 to 7.48 percent (*p* < 0.001) and in fasting glucose from 152.41 to 137.16 mg/dL (*p* < 0.001). In the subgroup analysis of CGM effectiveness, fasting glucose reduction was greater in females than in males and in patients with type 2 diabetes than in those with type 1 diabetes. In conclusion, it is essential to consider patient characteristics to enhance the effectiveness of CGM and to expand its use to type 2 diabetes to reduce the social burden of the disease.

## 1. Introduction

Type 2 diabetes mellitus (T2DM), commonly referred to as one of the modern lifestyle diseases, is characterized by elevated blood glucose levels resulting from increased insulin resistance and impaired function of insulin and other glucose-regulating hormones [1]. With the advent of an aging society, the prevalence of T2DM has steadily increased, posing significant public health concerns not only due to the disease itself but also because of its associated complications, including diabetic retinopathy, peripheral neuropathy, foot ulcers, and atherosclerosis [2,3].

Patients with T2DM have a threefold higher risk of developing cardiovascular disease compared to individuals of the same sex and age group with normal glucose tolerance [4]. Furthermore, recent evidence indicates that glycemic variability is strongly associated with diabetes-related complications and even increased mortality [5,6]. The pathophysiology of T2DM involves both insulin resistance and a progressive decline in insulin secretion, ultimately leading to chronic hyperglycemia. Therefore, optimizing glycemic control remains a central goal in T2DM management [7].

Approximately 589 million adults aged 20 to 79 years are living with diabetes globally, and this number is projected to rise to 853 million by 2050, nearly doubling in prevalence [8]. Individuals with diabetes are recommended to monitor their blood glucose regularly, follow a structured diet and exercise regimen, and consult healthcare professionals to maintain their disease and health. Continuous monitoring of blood glucose levels is crucial in this regard [9].

**Figure 1:**
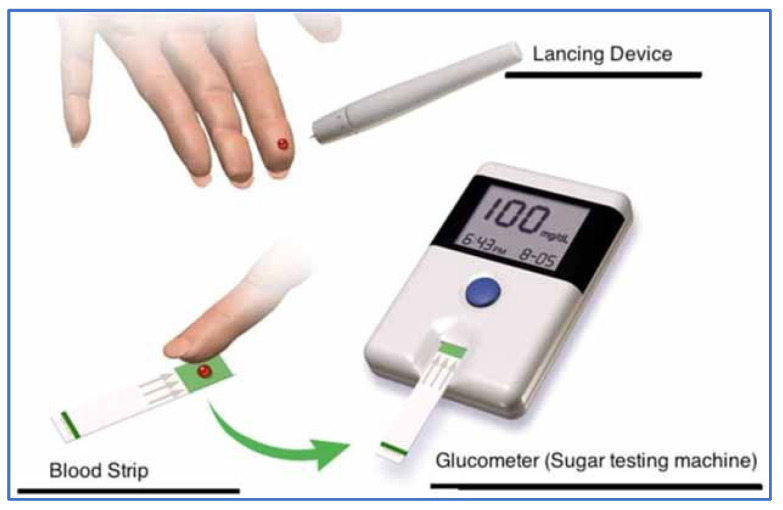
SMBG monitor.

Until recently, self-monitoring of blood glucose (SMBG) using fingerstick methods, shown in Figure 1, was the standard approach. However, SMBG poses several limitations, including the inconvenience of frequent finger pricks, associated pain, and limited capacity for long-term glycemic tracking [10,11]. Particularly among patients with type 1 diabetes, SMBG cannot detect real-time fluctuations, rendering them more vulnerable to acute complications such as hypoglycemic shock and diabetic ketoacidosis [12].

To address these limitations, real-time continuous glucose monitoring devices, which eliminate the need for blood sampling and allow for real-time monitoring, have been introduced since the mid-2010s following FDA approval. CGM devices, which involve placing a sensor on the skin to continuously measure glucose levels, offer significant advantages over traditional methods by capturing glycemic patterns and trends in addition to point measurements, thereby facilitating more stable glucose management [13,14]. Furthermore, while conventional glucose meters require consumables such as test strips and blood samples, CGM devices, as wearable technology, enable simplified, continuous measurement without consumable constraints [15].

By using CGM devices with high reliability and lower user burden, patients can more promptly detect and respond to glycemic fluctuations, thereby reducing disease-related stress and improving both physical and psychological well-being [16]. In Korea, the regulatory environment has evolved since 2016–2017, with multiple CGM products obtaining CE marking and FDA approval, enabling access through international purchase. As of 2023, CGM devices are classified as class III medical devices under the item code A26110.02 (“Personal Implantable Continuous Glucose Monitoring Systems”), which indicates a relatively high-risk category. To distribute such devices, manufacturers must submit clinical evidence and an official application to the Ministry of Food and Drug Safety [17].

Since 2019, CGM has been covered under national health insurance for patients with type 1 diabetes in Korea. In response, the number of type 1 diabetic patients using CGM devices has increased significantly [18]. In addition, the 7th edition of the Korean Diabetes Association’s clinical practice guidelines, published in 2021, included new recommendations for CGM use [19], further supporting its adoption. However, patients with type 2 diabetes are still not eligible for reimbursement under the current system. Given this gap, it is necessary to evaluate the effects of CGM use specifically among patients with type 2 diabetes.

This study aimed to investigate the effectiveness of CGM in improving self-management capacity and sociodemographic and health behavioral factors on glycemic control among diabetes patients using CGM. The specific objectives of this study are as follows:

- To identify the general characteristics of patients with diabetes who used CGM;
- To evaluate the change in glycemic (HbA1c and fasting glucose) and metabolic (BMI and total cholesterol) indicators before and after CGM use;
- To examine whether the effectiveness of CGM differs according to socio-demographic and health behavioral characteristics of diabetic patients.

## 2. Materials and Methods

### 2.1. Study Design

This study employed a single-group pretest-posttest experimental design to evaluate the effectiveness of the CGM system [20]. Patients diagnosed with diabetes at the Department of Endocrinology of K Hospital were instructed to self-manage their condition using the CGM system. The objective was to assess changes in outcomes before and after CGM use within the same cohort [21] (Table 1).

Inclusion criteria consisted of adults aged 18 years or older with a diagnosis of type 1 or type 2 diabetes mellitus according to American Diabetes Association (ADA) criteria, initiation or prior use of continuous glucose monitoring (CGM) for at least 12 weeks, and the ability to comply with study procedures. Exclusion criteria included pregnancy or planned pregnancy during the study period, participation in another interventional trial or use of investigational drugs within the past three months, severe comorbidities that could affect metabolic outcomes, known allergy or intolerance to CGM sensors or adhesives, and/or inability to perform CGM or self-monitoring due to cognitive impairment or other investigator-determined reasons.

### 2.2. Theoretical Background

#### 2.2.1. Glycated Hemoglobin (HbA1c)

HbA1c levels were analyzed using the BIO-RAD D-100 analyzer (Bio-Rad Laboratories, Inc., Hercules, CA, USA), which is employed in the diagnostic laboratory of K tertiary hospital in Seoul. HbA1c represents the proportion of glucose bound to hemoglobin and serves as a key blood marker for assessing long-term glycemic control over the preceding 2 to 3 months. It is widely used for both the diagnosis and management of diabetes [22].

#### 2.2.2. Fasting Plasma Glucose (FPG)

Several indices are commonly used to assess blood glucose management, including postprandial glucose, HbA1c, and fasting plasma glucose (FPG). FPG refers to the concentration of glucose in the blood following a fasting period of approximately 8 to 12 h [23]. Among patients with diabetes, elevated FPG levels are considered as important as postprandial glucose levels, as high FPG is significantly associated with a range of adverse health outcomes, including diabetes-related complications, cardiovascular disease, chronic kidney disease, and various types of cancer conditions that collectively contribute to a substantial global disease burden [24].

#### 2.2.3. Body Mass Index (BMI)

Body mass index (BMI), calculated by dividing an individual’s weight in kilograms by the square of their height in meters (kg/m^2^), is a widely used indicator of obesity as it reflects body fat composition with relatively high accuracy [25]. In this study, BMI was categorized into three groups: low weight (<18.5), normal weight (18.5–24.9), and overweight (≥25.0) [26]. Measurements were obtained using the BSM 370 device, which is routinely used in the endocrinology laboratory at K tertiary hospital in Seoul.

#### 2.2.4. Total Cholesterol

Elevated levels of triglycerides and cholesterol have been shown to be significantly associated with increased blood glucose concentrations [27]. Among lipid parameters, total cholesterol is a critical factor influencing the prognosis of patients with T2DM. Elevated total cholesterol levels are linked to a heightened risk of coronary heart disease, a major contributor to morbidity and mortality in individuals with T2DM. In addition, inadequate glycemic control exacerbates both microvascular and macrovascular complications. Accordingly, regular monitoring and appropriate management of total cholesterol are essential to reducing the risk of life-threatening complications in T2DM patients [28].

#### 2.2.5. Continuous Glucose Monitoring System

A CGM system typically consists of a sensor that measures glucose levels in the interstitial fluid beneath the skin, a transmitter that sends the measured data, and a monitor that receives and displays the transmitted values [29]. In this study, the intermittently scanned continuous glucose monitoring (isCGM) system was utilized [30], in which the sensor continuously records glucose levels but requires the user to initiate a scan to access the information. It does not require user-performed blood glucose calibration, allowing for continuous use over a 14-day period.

### 2.3. Ethical Considerations for Participants

To ensure the protection of personal information and data security, patient data obtained from the electronic medical record (EMR) system of individuals treated for diabetes were de-identified. All personal identifiers were removed and replaced with placeholder values to maintain anonymity. The study was conducted in accordance with the Declaration of Helsinki and approved by the Institutional Review Board of B University (IRB No. 2-7008132-A-N-01. 25-11).

### 2.4. Data Collection and Statistical Analysis

Data were collected through a pre-test administered during the CGM education session at the initial cohort visit, followed by a post-test at the final visit 12 weeks later. Data were analyzed using SPSS version 27 (IBM SPSS Statistics 27, IBM Corporation, Armonk, NY, USA). Descriptive statistics for participants’ general characteristics were presented as frequencies and percentages. Paired sample *t*-tests were conducted to compare changes in glycemic and metabolic indicators before and after the use of CGM. Independent sample *t*-tests were used to examine differences in changes in BMI and HbA1c according to age groups. Multiple linear regression analysis was conducted to estimate the effect sizes of factors influencing glycemic variability associated with CGM use. All statistical tests were performed with a significance level of 5% [31].

## 3. Results

### 3.1. General Characteristics of Study Subjects

Table 2 presents the sociodemographic and health-related characteristics of subjects including diabetes type, smoking behavior, and alcohol consumption. A total of 510 participants were included in the study, comprising 325 males (63.7%) and 185 females (36.3%). Based on age, 316 participants (62.0%) were under 65 years old, while 194 (38.0%) were aged 65 years or older. Regarding body mass index (BMI), 11 participants (2.2%) were classified as underweight (BMI < 18.5), 244 (47.8%) had a normal BMI (18.5–24.9), and 255 (50.0%) were classified as overweight (BMI ≥ 25.0). In terms of diabetes type, 13 participants (2.5%) were diagnosed with type 1 diabetes, while 497 (97.5%) had type 2 diabetes.

### 3.2. Changes in Glycemic and Metabolic Indicators Associated with the Use of CGM

Paired sample *t*-tests were conducted to compare the mean values of four outcomes, including HbA1c, fasting glucose, BMI, and total cholesterol, between pre- and post-intervention measurements. The results showed a significant reduction in HbA1c from 8.09 to 7.48 (*p* < 0.001), and fasting glucose also significantly decreased from 152.41 to 137.16 (*p* < 0.001). Although BMI increased slightly from 25.16 to 25.64, this change was not statistically significant (*p* = 0.323). Total cholesterol decreased significantly from 149.77 to 146.95 (*p* = 0.036) (Table 3).

### 3.3. Differences in CGM Effects on HbA1c According to Sociodemographic and Health Behavioral Characteristics

The differences in changes in HbA1c levels before and after the use of continuous glucose monitoring (CGM) were analyzed using *t*-tests and one-way ANOVA according to sex, age group, body mass index (BMI), type of diabetes, smoking status, and alcohol consumption (Table 4).

By sex, the reduction in HbA1c was greater in males (−0.67%) than in females (−0.50%); however, this difference was not statistically significant (*p* = 0.158). When compared by age group, adults aged under 65 showed a slightly greater reduction (−0.64%) than older adults aged 65 and above (−0.57%), but the difference was not statistically significant (*p* = 0.590). According to BMI categories, individuals with normal weight showed the greatest decrease in HbA1c (−0.68%), followed by those classified as obese (−0.55%) and underweight (−0.51%), though these differences were not statistically significant (*p* = 0.525). When comparing the change in HbA1c by diabetes type, patients with type 2 diabetes experienced a reduction of −0.62%, while those with type 1 diabetes showed a smaller reduction of −0.14%; however, this difference was not statistically significant (*p* = 0.198). Regarding smoking status, non-smokers demonstrated the greatest reduction in HbA1c (−0.93%), followed by former smokers (−0.61%) and current smokers (−0.57%), though these differences did not reach statistical significance (*p* = 0.224). Lastly, in terms of alcohol consumption, former drinkers showed the most pronounced reduction in HbA1c (−1.63%), compared to current drinkers (−0.61%) and non-drinkers (−0.51%), but again, the differences were not statistically significant (*p* = 0.275).

### 3.4. Differences in CGM Effects on Fasting Blood Glucose According to Sociodemographic and Health Behavioral Characteristics

Independent *t*-tests and one-way ANOVA were conducted to examine whether changes in fasting glucose levels before and after CGM use differed according to sex, age group, body mass index (BMI), type of diabetes, smoking status, and alcohol consumption. Table 5 shows that the reduction in fasting glucose was significantly greater in males (−19.67 mg/dL) than in females (−7.49 mg/dL) (*p* = 0.024). Older adults showed a slightly greater decrease (−15.34 mg/dL) compared to younger adults (−15.20 mg/dL), but this difference was not statistically significant (*p* = 0.980). In terms of BMI, reductions in fasting glucose were observed in the normal weight (−18.21 mg/dL), underweight (−13.27 mg/dL), and obesity (−12.51 mg/dL) groups, though the differences were not statistically significant (*p* = 0.555). Regarding diabetes type, patients with type 2 diabetes experienced a significant decrease in fasting glucose (−16.32 mg/dL), whereas those with type 1 diabetes showed an increase (+25.46 mg/dL), with the difference being statistically significant (*p* = 0.011). For smoking status, reductions were observed in former smokers (−22.83 mg/dL), current smokers (−16.39 mg/dL), and non-smokers (−14.02 mg/dL); however, these differences were not statistically significant (*p* = 0.556).

### 3.5. Factors Influencing Fasting Glucose Variability Associated with Continuous Glucose Monitoring

Multiple regression analysis was conducted to assess the effects of sex, age, body mass index (BMI), type of diabetes, and smoking status on changes in fasting glucose following the use of the CGM system (Table 6). To establish a statistically significant multiple linear regression model, the variable for alcohol consumption was excluded from the analysis. Although the explanatory power of the regression model was relatively low (R^2^ = 0.026), the model was statistically significant (*p* = 0.038), indicating that it possessed predictive validity.

Regarding sex, female patients experienced a significantly smaller reduction in fasting glucose compared to males, with a mean difference of 11.78 mg/dL (*p* = 0.041). For age, fasting glucose tended to decrease by 0.21 mg/dL for each additional year of age, though this association was not statistically significant (*p* = 0.398). With respect to BMI, a one-unit (kg/m^2^) increase was associated with a 0.78 mg/dL smaller decrease in fasting glucose; however, this relationship was also not statistically significant (*p* = 0.283). In terms of diabetes type, patients with type 1 diabetes showed a 47.82 mg/dL smaller reduction in fasting glucose compared to those with type 2 diabetes, and this difference was statistically significant (*p* = 0.006). As for smoking status, current smokers showed a smaller reduction in fasting glucose (by 4.33 mg/dL) compared to non-smokers, but this difference was not significant (*p* = 0.647). Former smokers showed a tendency toward a greater reduction (by 2.70 mg/dL) compared to non-smokers, although this difference was also not statistically significant (*p* = 0.751).

## 4. Discussion

A previous study evaluating the effectiveness of CGM use in reducing glycated hemoglobin (HbA1c) demonstrated a 0.55% reduction in HbA1c levels after 2 to 4 months of CGM use [32,33], which is comparable to the 0.61% reduction in HbA1c observed in this study.

There was previous research reporting that the glycemic control effect of CGM was more prominent in females and in individuals with lower baseline HbA1c levels [34]. However, this contrasts with the findings of this study, in which the reduction in fasting glucose following CGM use was greater in males (−19.67 mg/dL) than in females (−7.49 mg/dL). These contrasting results highlight the need for further research to clearly identify the differences in CGM effectiveness based on sociodemographic factors such as sex, age, education, and income level.

The importance of real-time glucose monitoring is increasingly emphasized, particularly given that diabetes is a lifelong chronic condition requiring sustained management. The adoption of CGM devices has contributed to improved quality of life for many patients by enabling a better understanding of glycemic fluctuations in daily life and facilitating more effective glucose management [35,36]. CGM is now considered a standard of care for chronic diabetes, and its widespread prescription underscores the necessity of evaluating patients’ lifestyle behaviors [37]. When integrated with other health-related data, CGM data can be utilized more effectively, supporting the development of data-driven personalized treatment approaches [38]. Such strategies are expected to enhance treatment efficacy, prevent complications, and improve patients’ quality of life.

This study demonstrated a statistically significant reduction in total cholesterol following CGM use (149.77 to 146.95 mg/dL). According to a prior clinical trial evaluating the Dexcom G6 CGM (Dexcom, Inc., San Diego, CA, USA) device in patients with type 2 diabetes mellitus not receiving insulin therapy, CGM use was associated with significant improvements in glycemic control as well as multiple cardiometabolic risk factors, including reductions in BMI, triglycerides, blood pressure, and total cholesterol [39]. Although differences exist between the study populations and intervention durations, the concordance in total cholesterol reduction across both studies strengthens the evidence that CGM may serve as an effective strategy not only for optimizing glycemic management but also for mitigating broader cardiovascular risk in patients with type 2 diabetes mellitus.

To maximize the benefits of CGM, it is necessary to establish educational reimbursement policies that ensure the quality of patient education while alleviating the burden on healthcare providers [40]. Additionally, insurance coverage should be expanded to support CGM-related education for patients with type 2 diabetes, as education plays a crucial role in facilitating CGM use [41]. From both clinical and educational perspectives, enhancing patients’ self-management capabilities through education on CGM use, data interpretation, and understanding glycemic patterns is essential. This includes individualized dietary counseling, medication adherence support, and physical activity guidance [42].

Our study found that patients with type 2 diabetes experienced a greater reduction of −0.62% in HbA1c compared to those with type 1 diabetes (−0.14%), which is consistent with a previous study reporting a more substantial decrease in HbA1c among type 2 diabetes patients using CGM [13]. However, in South Korea, continuous glucose monitoring devices have been covered by national health insurance only for patients with type 1 diabetes since 2020, whereas coverage has not yet been extended to those with type 2 diabetes due to concerns about increasing national healthcare expenditures [43]. Nonetheless, there is a growing demand for the inclusion of CGM under insurance coverage for type 2 diabetes patients. The latest Standards of Care 2025 from the ADA recommend the use of CGM as beneficial for improving glycemic control, not only in individuals with type 2 diabetes who use insulin, but also in those who do not use insulin [44].

In this study, BMI did not change significantly after CGM use, which is consistent with previous studies reporting that no significant correlations were found between BMI and other CGM metrics as the weight loss or BMI is more possible when lifestyle and therapeutic auxiliary factors such as nutritional counseling, exercise, and medication are combined, rather than being specified as the expected effects of CGM alone [45,46].

This study provides empirical evidence that CGM use in diabetes management extends beyond simple glucose monitoring and enhances self-management capacity. These findings offer a foundation for future research and clinical practice, validating the effectiveness of CGM in promoting glycemic regulation through improved self-care behaviors.

The study findings highlight the practical value of CGM in routine diabetes care by demonstrating meaningful improvements in key glycemic indicators (HbA1c and fasting glucose) and metabolic parameters over only 12 weeks of use in a real-world cohort. These results reinforce CGM not merely as a monitoring tool but as an intervention that can actively support tighter glycemic control and reduce future cardiovascular risk factors [47]. Importantly, the subgroup differences with greater fasting-glucose reduction in females and in patients with type 2 diabetes suggest that patient characteristics substantially influence CGM effectiveness and should be taken into account when selecting candidates, tailoring education, and planning reimbursement or guideline expansion [48]. By targeting populations most likely to benefit, clinicians and policymakers may enhance treatment efficiency, improve long-term outcomes, and ultimately lessen the societal and economic burden of diabetes.

Despite the significance of the research, this study has several limitations. First, there is a potential risk of bias due to the study design, which lacked a control group. Second, the study duration was three months, which may have been insufficient either for the full effect of CGM use to manifest or for assessing any attenuation of efficacy as participants became accustomed to the CGM system. Third, the potential confounding variables were not comprehensively measured when evaluating the effects of CGM use on glycemic control and metabolic health outcomes. As a result, unmeasured or residual confounding may have influenced the observed associations. Future studies should include a more thorough assessment of relevant confounders to strengthen the validity of the findings. Lastly, there is a limitation in generalizing the findings regarding differences in CGM effectiveness between the T1DM and T2DM groups, as only 13 participants (2.5%) with T1DM were included compared with 497 participants (97.5%) with T2DM.

## 5. Conclusions

Optimizing the clinical benefits of CGM requires proactive assessment of patients’ sociodemographic characteristics to enable tailored interventions that maximize its impact. Given the growing evidence of its utility, expanding CGM use beyond individuals with type 1 diabetes to include those with type 2 diabetes is a critical step toward improving glycemic control and overall metabolic health. To facilitate this expansion, healthcare policies should prioritize reducing financial barriers through insurance coverage or reimbursement programs, thereby ensuring equitable access to CGM and fostering broader adoption in routine diabetes care. Future research should focus on developing integrated care models and long-term outcome studies to evaluate the sustained impact of CGM on metabolic parameters, patient-reported outcomes, and healthcare costs, as well as on leveraging emerging technologies such as digital coaching and predictive analytics to further enhance CGM’s effectiveness.

## Figures and Tables

**Table 1:** Study design.

Group	Pre-Test	→	Treatment or Intervention	→	Post-Test
All subjects	O_1_		X_2_		O_3_

O_1_: Baseline test for HbA1c, fasting glucose, BMI, and total cholesterol. X_2_: Blood glucose management using continuous glucose monitoring system. O_3_: Post-intervention test for HbA1c, fasting glucose, BMI, and total cholesterol.

**Table 2:** Sociodemographic and health-related characteristics of study subjects (*n* = 510).

Variable	*n* (%)	Mean ± SD
Gender		
Male	325 (63.7)	
Female	185 (36.3)	
Age ^1^		59.71 ± 11.37
<65	316 (62.0)	
≥65	194 (38.0)	
BMI ^2^		25.12 ± 3.87
Low weight (<18.5)	11 (2.2)	
Normal (18.5–24.9)	244 (47.8)	
Obesity (≥25)	255 (50.0)	
Diabetes type		
Type 1	13 (2.5)	
Type 2	497 (97.5)	
Smoking		
Non-smoking	405 (79.4)	
Current-smoking	46 (9.0	
Ex-smoking	59 (11.6)	
Drinking		
Non-drinker	450 (88.2)	
Current-drinker	56 (11.0)	
Ex-drinker	4 (0.8)	

SD: standard deviation. ^1^ Units expressed as years. ^2^ Units expressed as kg/m^2^.

**Table 3:** Effect of continuous glucose monitoring on glycemic variability (*n* = 510).

Variable	Baseline	Post-CGM	t	*p*-Value
	Mean ± SD	Mean ± SD		
HbA1C ^1^	8.09 ± 0.06	7.48± 0.05	10.297	<0.001
Fasting glucose ^2^	152.41 ± 2.54	137.16 ± 1.85	5.861	<0.001
BMI ^3^	25.16 ± 0.17	25.64 ± 0.51	−0.990	0.323
Total cholesterol ^4^	149.77 ± 1.66	146.95 ± 1.53	2.108	0.036

SD: standard deviation. ^1^ Units expressed as percent. ^2^ Units expressed as mg/dL. ^3^ Units expressed as kg/m^2^. ^4^ Units expressed as mg/dL.

**Table 4:** Subgroup Analysis of CGM Effects on HbA1c according to Sociodemographic and Health Behavioral Characteristics (*n* = 510).

Variable	HbA1c Variation	t, F	*p*-Value
	Mean ± SD		
Gender		−1.412	0.158
Male	−0.67 ± 1.45		
Female	−0.50 ± 1.12		
Age ^1^		−0.539	0.590
<65	−0.64 ± 1.42		
≥65	−0.57 ± 1.21		
BMI ^2^		0.645	0.525
Low weight (<18.5)	−0.51 ± 0.91		
Normal (18.5–24.9)	−0.68 ± 1.28		
Obesity (≥25)	−0.55 ± 1.41		
Diabetes type		1.289	0.198
Type 1	−0.14 ± 1.03		
Type 2	−0.62 ± 1.35		
Smoking		1.502	0.224
Non-smoking	−0.93 ± 1.75		
Current-smoking	−0.57 ± 1.29		
Ex-smoking	−0.61 ± 1.27		
Drinking		1.294	0.275
Non-drinker	−0.51 ± 1.56		
Current-drinker	−0.61 ± 1.31		
Ex-drinker	−1.63 ± 1.24		

SD: standard deviation. ^1^ Units expressed as years. ^2^ Units expressed as kg/m^2^.

**Table 5:** Subgroup Analysis of CGM Effects on Fasting Blood Glucose according to Sociodemographic and Health Behavioral Characteristics (*n* = 510).

Variable	Fasting Glucose Variation	t, F	*p*-Value
	Mean ± SD		
Gender		−2.259	0.024
Male	−19.67 ± 61.66		
Female	−7.49 ± 52.58		
Age ^1^		0.025	0.980
<65	−15.20 ± 59.80		
≥65	−15.34 ± 57.20		
BMI ^2^		0.590	0.555
Low weight (<18.5)	−13.273 ± 37.40		
Normal (18.5–24.9)	−18.21 ± 60.46		
Obesity (≥25)	−12.51 ± 57.91		
Diabetes type		2.544	0.011
Type 1	25.46 ± 60.06		
Type 2	−16.32 ± 58.42		
Smoking		0.587	0.556
Non-smoking	−14.02 ± 60.43		
Current-smoking	−16.39 ± 58.46		
Ex-smoking	22.83 ± 46.42		
Drinking		0.173	0.842
Non-drinker	−19.34 ± 71.17		
Current-drinker	−14.70 ± 57.20		
Ex-drinker	−20.75 ± 55.51		

SD: standard deviation. ^1^ Units expressed as years. ^2^ Units expressed as kg/m^2^.

**Table 6:** Multiple Linear Regression Analysis for Fasting Blood Glucose Variance associated with CGM use (*n* = 510).

Variable		B	S.E	β	t	*p*-Value	VIF
(Constant)		−52.65	26.26		−2.005	0.045	
Gender	Female	11.78	5.76	0.10	2.045	0.041	1.15
Age (years)		0.21	0.24	0.04	0.846	0.398	1.15
BMI (kg/m^2^)		0.78	0.72	0.05	1.076	0.283	1.09
Diabetes type	Type 1	47.82	17.23	0.13	2.776	0.006	1.10
Smoking	Current smoker	4.33	9.46	0.02	0.458	0.647	1.10
	Ex-smoker	−2.70	8.52	−0.01	−0.317	0.751	1.11
R^2/^Adjusted R^2^		0.026/0.014					
F(df), *p*-value		2.238(6), 0.038					

## Data Availability

The data presented in this study are available on request from the corresponding author.
